# Facilitating large-scale implementation of evidence based health care: insider accounts from a co-operative inquiry

**DOI:** 10.1186/s12913-015-0722-6

**Published:** 2015-02-13

**Authors:** Heather Waterman, Ruth Boaden, Lorraine Burey, Brook Howells, Gill Harvey, John Humphreys, Katy Rothwell, Michael Spence

**Affiliations:** School of Nursing, Midwifery and Social Work, Jean McFarlane Building, University of Manchester, Oxford Road, Manchester, M13 9PL UK; Manchester Business School, University of Manchester, Booth St. West, Manchester, M15 6 PB UK; CLAHRC, 3rd Floor Mayo Building, Salford Royal NHS Foundation Trust, Stott Lane, Salford, M6 8HD UK; Manchester Business School, University of Manchester, Booth St. West, Manchester, M15 6 PB UK; School of Nursing, University of Adelaide, Adelaide, Australia; Advancing Quality Alliance, Salford Royal NHS Foundation trust, Stott Lane, Salford, M6 8HD UK

**Keywords:** Evidence based practice, Facilitation, Facilitators, Co-operative inquiry, Evidence based health care, Utilization of research, Implementation of research

## Abstract

**Background:**

Facilitators are known to be influential in the implementation of evidence-based health care (EBHC). However, little evidence exists on what it is that they do to support the implementation process. This research reports on how knowledge transfer associates (KTAs) working as part of the UK National Institute for Health Research ‘Collaboration for Leadership in Applied Health Research and Care’ for Greater Manchester (GM CLAHRC) facilitated the implementation of EBHC across several commissioning and provider health care agencies.

**Methods:**

A prospective co-operative inquiry with eight KTAs was carried out comprising of 11 regular group meetings where they reflected critically on their experiences. Twenty interviews were also conducted with other members of the GM CLAHRC Implementation Team to gain their perspectives of the KTAs facilitation role and process.

**Results:**

There were four phases to the facilitation of EBHC on a large scale: (1) Assisting with the decision on what EBHC to implement, in this phase, KTAs pulled together people and disparate strands of information to facilitate a decision on which EBHC should be implemented; (2) Planning of the implementation of EBHC, in which KTAs spent time gathering additional information and going between key people to plan the implementation; (3) Coordinating and implementing EBHC when KTAs recruited general practices and people for the implementation of EBHC; and (4) Evaluating the EBHC which required the KTAs to set up (new) systems to gather data for analysis. Over time, the KTAs demonstrated growing confidence and skills in aspects of facilitation: research, interpersonal communication, project management and change management skills.

**Conclusion:**

The findings provide prospective empirical data on the large scale implementation of EBHC in primary care and community based organisations focusing on resources and processes involved. Detailed evidence shows facilitation is context dependent and that ‘one size does not fits all’. Co-operative inquiry was a useful method to enhance KTAs learning. The evidence shows that facilitators need tailored support and education, during the process of implementation to provide them with a well-rounded skill-set. Our study was not designed to demonstrate how facilitators contribute to patient health outcomes thus further prospective research is required.

## Background

Facilitation to support research utilization for the benefit of patients and clients has grown in significance over the past twenty years. This is in contrast to passive methods of disseminating research, which are seen as insufficient in influencing its uptake [1]. However, questions remain unanswered as to how facilitation is actually achieved in practice.

### Definition and concept

The concept of facilitation in the context of evidence based health care (EBHC) has been defined as: ‘a technique by which one person makes things easier for others’ p152 [2]. Facilitation can positively influence the uptake of research [3,4]. A quantitative synthesis of 23 controlled studies shows that the overall effect size for practice facilitation on the adoption of evidence based guidelines is moderate (2.76 times more likely) but identifies that fidelity factors, number of general practices per facilitator and intensity of intervention modify the effect [5]. However, a conceptual analysis found there is little empirical research on what is involved in facilitation including the relative importance of styles of facilitation, how facilitation skills can be positively honed and which components of the facilitation process or which roles are most effective and why [6].

To our knowledge, there is one qualitative research study that explicitly focused on the facilitation of large-scale EBHC implementation, which consists of retrospective interviews with external facilitators of a central change agency, the Quality Enhancement Research Initiative [7]. The findings suggest these facilitators assisted internal change agents to realise local implementation of EBHC using interactive problem solving, education and support [7]. A prospective, critically reflective study of facilitation will provide further insight into how and why facilitation unfolds, what methods are used by facilitators and how they overcome contextual problems which may be holding back implementation. This method seeks to share experiences among those in a similar situation to develop their understanding and skills through an on-going iterative process of action and reflection. Thus, analysis of this process offers useful insight into what they do and learn.

Facilitation may be employed as both a noun, that is, ‘facilitator’ and as a verb, ‘to facilitate’ [8]. This is problematic when attempting to evaluate facilitation because unless clearly stated it leads to confusion surrounding what is being evaluated. For purposes of clarification, we employ facilitation in the latter sense; knowledge transfer associates (KTAs) facilitated the implementation of EBHC. They were not called ‘facilitators’ but the process and activities of facilitation were examined.

As indicated, there are many competing concepts that employ facilitative approaches including change agents, opinion leaders, KTAs, knowledge brokers. Several of the more common roles for the implementation of EBHC are presented in Table 1 according to specific criteria [1]. As originally identified, even though the roles have different underpinning theoretical perspectives, they appear to employ the same facilitation processes and activities. This only serves to create confusion surrounding which concepts and terms to employ making comparisons between studies difficult [1].

**Table 1:** **Comparison of roles that facilitate the implementation of EBHC**

**Criteria**	**Opinion Leader**	**Facilitator**	**Champion**	**Change agent**	**Knowledge broker**	**Knowledge transfer associate KTA**
Theory	Social influence	Problem solving models	Social influence	Change theory	Rational linear model of EBHC	The PARIHS model (Promoting action on research implementation, Kitson et al., [2]);
Specific purpose	Evaluate	Achieve change/goal	Promote	Change behaviour	Implement research into health care	Implement research into health care
Role	Informal	Formal	Informal	Formal	Formal	Formal
Nature of evidence	Expert, experiential	Collective construction	Expert, experiential	Rational, experiential, expert	Rational	Integrated approach - theoretical, experiential, contextual and empirical
Who are they?	Individual	Individual	Individual or organisational	Individual or organisational	Individual or Organisational	Individual and Organisational
Trained or employed for role	No	Yes	No	Yes	Yes	Yes
Domain of influence	Work unit, speciality	Boundary spanning	Project specific	Boundary spanning	Boundary spanning	Boundary spanning
Relationships	On-going	Short-term	On-going	Short-term	Short-term	Short-term
Organisational orientation	Internal	Internal or external	Internal	Internal or external	Internal or External	Internal or External

(Adapted from Thompson et al., [1]).

### Frameworks for EBHC and underpinning paradigms

There are also numerous models and frameworks providing an overview and explanation of the process of the implementation of EBHC. The older frameworks tend to draw on a rationalistic approach defining research implementation as a technical one-way process [9]. More recent frameworks draw on a constructionist approach arguing that the social context of structures, norms, power, ideologies, culture and human action interact in complex ways to influence how and whether EBHC is implemented [10,11]. For this project, we took the view that a rational linear perspective was inadequate, that we would be responsive to the context, and we would recognise that relationships especially power relations, would be important factor in mediating research [12].

### Definition of EBHC

A popular definition of EBHC is ‘the conscientious use of current best evidence in making decisions about the care of individual patients or the delivery of health services’ [13]. From the biomedical tradition, level one evidence on the effectiveness of a health technology intervention equates with controlled, clinical trials or systematic reviews thereof [14]. However, findings from implementation studies suggest that a broader definition of evidence incorporating theoretical ideas and evidence from experience is actually employed in practice [15]. For the purposes of this study, a broad definition of evidence is employed, referring not only to empirical but also to theoretical and experiential evidence [12]. This broad definition is also used widely in the preparation of clinical guidelines, for example, by the GRADE method, whereby clinical and patient experiences contribute to the evidence base, alongside empirical research [16]. EBHC in this study refers to the innovations or changes made in light of evidence.

### Implementing EBHC in practice: the greater Manchester CLAHRC

The Greater Manchester ‘Collaboration for Leadership in Applied Health Research and Care’ (GM CLAHRC) is i) funded by the Department of Health National Institute for Health Research (NIHR) and ii) by matched funding from local National Health Service (NHS) partners in the UK [12]. The GM CLAHRC has responsibility for conducting high quality applied research and implementation of research into practice to enhance patient benefit in the NHS. The KTA role for the GM CLAHRC was adapted from the Knowledge Transfer Partnerships (KTP) model [17]. Each KTA was part of a team responsible for implementing EBHC projects related to the management of patients with long term conditions representing priority public health issues (Table 2). They worked under the guidance of a clinical lead (CL), an academic lead (AL) and programme manager (M) who had expertise in change management or other related fields [12]. These projects were based in primary- and community-care involving 20 NHS organisations serving a combined population of 2.55 million.

**Table 2:** **The six initial projects of the Greater Manchester CLAHRC**

Stroke	Implementation of six month post-stroke reviews (a requirement of the National Stroke Strategy)*.
Diabetes	Implementation of an intensive lifestyle intervention service for people at risk of developing type 2 diabetes.
Chronic kidney disease	Implementation of early identification and management systems of people with early stage kidney disease.
Heart Failure Alert Cards	Implementation of patient held discharge cards for the improvement of communication and transition of care between primary, community and secondary organisations.
Heart Failure	Implementation of a programme of education and management systems to raise awareness and improve clinical skills.
Heart Failure Website	Implementation of heart failure website to bring together resources for patients with heart failure and for those who care for them.

*Department of Health. National Stroke Strategy. London: Department of Health, 2005.

When the GM CLAHRC began, the KTAs had little direct knowledge of how to implement EBHC, or of the clinical conditions to which their projects pertained except for one KTA who was a nurse by background. Several approaches were employed to assist the KTAs to help them understand and deal with the complexities and difficulties of implementing EBHC including formal teaching sessions and a co-operative inquiry. This paper focuses on the co-operative inquiry. The aims of the co-operative inquiry were: first, that through the process of co-operative inquiry the KTAs would understand better how to implement EBHC, and, second, to provide a qualitative descriptive account of how they facilitated, at both a commissioner and provider level, the implementation of EBHC.

## Methods

### Design and participants

The KTAs needed to understand how to facilitate the implementation of EBHC across many organisations and commissioning agencies. ‘Co-operative inquiry’ was selected as the research design because it is a way of researching with people who have related experiences, and who wish to examine with others how they might extend and deepen their understanding of their situation, and to learn how to improve their actions [18]. As in other forms of action research, research participants in co-operative inquiry are active associates who participate in the ‘thinking’ and the ‘doing’ of the research. So in this project, the KTAs made most of the research decisions and carried out part of the research.

The initiating researcher, HW, played the role of facilitator who enabled candid and non-judgmental discussions among the KTAs and assisted them in developing a critical attitude to their reflections. She was deemed a co-researcher alongside the KTAs as she too, although she was not working directly to facilitate EBHC, wished to improve the process of facilitation. HW agreed to carry out interviews and do some of the data analysis with two research assistants because the KTAs could not afford the time. HW has twenty-two years’ experience of action research.

The KTAs and HW were both researchers and participants in a transformative research process so the usual notion of researcher neutrality and objectivity could not apply. A reflexive approach was therefore chosen to understand how past and on-going personal and professional experiences influenced the research [19]. During meetings, each person’s perspective was explored and critically analysed giving rise to new or expanded learning thus reducing the likelihood of unwarranted reinforcement of original biases.

Basically, the KTAs came together for reflective meetings to share what they did on a daily basis, to explore their successes and problems in detail, and to make sense of their experiences. After they would return to their projects and return a month later for further discussions and so on. The intensely reflective method of co-operative inquiry was considered by the KTAs to be pertinent to their educational needs and was an important reason why co-operative inquiry was chosen. Other critical collaborative research methods, such as critical ethnography, could have been chosen but participant self-development in co-operative inquiry was the crucial reason for its selection [20].

Co-operative inquiry has an extended epistemology of four types of knowing: practical (how to do something), experiential (direct encounter), propositional (theory) and presentational (stories) knowledge [21]. During the co-operative inquiry, all four types of knowledge were explored. This epistemological approach was attractive because it enabled the achievement of both of the proposed aims.

Research questions were negotiated and agreed by the KTAs:

1. How do KTAs facilitate the implementation of EBHC in the NHS to improve patient/client care?
2. How do the GM CLAHRC managers (Ms), clinical leads (CLs) and academic leads (ALs) perceive KTAs facilitate the implementation of EBHC?

### Procedure

A voluntary co-operative inquiry group was formed that consisted of the KTAs and HW. The inquiry passed through the following cyclical phases [21]:

- Phase 1: Preliminary meetings to agree a plan for the inquiry
- Phase 2: Provisional sense making by the KTAs as co-subjects
- Phase 3: KTAs immerse and engage with their action and experience
- Phase 4: The original plan and sense making are reviewed by KTAs in light of phases 2 and 3

Repeat phases 2 and 3 and so on until agreement to finish.

*Phase 1:* Over six months, the KTAs agreed the research design, who would be involved, and the method of data collection and analysis. The agreed approach to data collection was qualitative because the focus of the study was to describe and understand how KTAs facilitate the implementation of EBHC [22]. Initially, two main methods of data collection were proposed: i) diaries of the KTAs’ experiences of facilitation and ii) audio-recordings of the co-operative inquiry meetings. Having gained a clear idea of the focus and structure of the inquiry, a research proposal was written and ethics approval sought. This study was approved by National Research Ethics Service Committee North West Preston (09/H1016/119).

*Phase 2:* Following informed consent, the KTAs met with HW on a regular, monthly basis. With respect to research question 1, the group first set out their original thinking about the topic under exploration. They chose to examine four different aspects of facilitation: i) How frameworks for the implementation of EBHC helped facilitation, ii) How relationships supported facilitation, iii) How they used evidence to influence change and iv) What factors influenced the facilitation approach. Then they ‘observed’ or ‘watched’ how they themselves facilitated the implementation of evidence in practice. This was then related and discussed in the group meetings. Despite careful preparation, KTAs found it difficult to fit writing diaries into their work load and so recordings of inquiry meetings became the only method of data collection. However, by consent these meetings lasted on average for two hours and allowed time for each KTA to recount their personal experiences.

*Phase 3:* KTAs became fully engaged with examining their experiences and actions. This meant the KTAs were more critical, open and flexible in their thinking than in Phase 2, which led to increasingly thorough discussions of their experiences.

Various techniques were employed to enhance the validity of findings. First, there is a danger that reflection may take precedence over action, which may make the research an ‘ivory tower’ exercise [23]. To counteract this, the meetings were held in parallel with the implementation of EBHC making the discussions grounded in action and where opinions were modified over time, as KTAs were able to reflect on their experiences and discussions with others. This also led to KTAs taking new or slightly different courses of action.

Second, conflict and open distress could have emerged during group meetings, which could have distorted the content of discussions. Possible scenarios for causes and solutions to conflict were discussed among the group so they were prepared for dealing with such situations, should they have arisen.

A third method to enhance validity is to develop authentic collaboration among the group. They did this by agreeing ground rules for interactions in the inquiry group, for example, by not talking over one another.

*Phase 4:* After four months of meetings, the KTAs met to compare their experiences with their original thinking. This led to a thorough review, expansion and refinement of their initial ideas. At this time, they began to realise that while they had been interested in four different aspects of facilitation, as outlined in Phase 2, their experience of these had altered over time. They changed their focus slightly therefore to examine the process of implementation. They then entered another cycle of action and reflection as described in Phase 3. The study stopped when saturation of data occurred.

A framework for the implementation of EBHC was not employed to structure their discussions because flexibility in discussions and freedom in analysis was preferred. However, operationally, GM CLAHRC employed project management processes so the KTA’s experience was imbibed with these.

To answer research question 2, semi-structured interviews were carried out with CLs (3), ALs (4) and Ms (5) at the beginning and end of the project to elicit, from their perspectives, how EBHC was facilitated. This helped to provide a check and balance to the interpretation of findings.

### Data analysis

All recordings of the KTAs inquiry meetings were transcribed using a University approved transcriber who was required to maintain confidentiality. Transcriptions were prepared according to an agreed format including, large margins and anonymous coding of interviewer and interviewee. Framework analysis was chosen as the method of analysis because it provides a transparent and structured approach while remaining grounded in the experiences of research participants [24]. Framework analysis begins with familiarisation with the data. In this case, this began four months into the project so that the draft analysis could be fed back at the KTA meetings for further critical reflection. Familiarisation consisted of reading, listening and gaining an overview of the data before it was reduced to smaller components from which key themes and their respective descriptors (categories) pertaining to the key phases of the process of facilitation were identified. This was carried out by HW. The preliminary thematic framework was shared with the KTAs where it was discussed, and insights and amendments provided. The transcripts were then coded (indexed) as per the thematic framework and the data was charted, with information from the transcripts placed into the thematic framework. This was carried out by HW with the assistance of two research assistants. Themes were reviewed by the KTAs from which two initial themes were collapsed. The framework that was developed reflects the four key stages of the process of large-scale implementation of EBHC experienced by the KTAs. Given that GM CLARHC used project management processes to drive forward change, it is unsurprising that the four stages are reminiscent of these. The analysis could have been structured according to the phases of the co-operative inquiry but in our approach the focus was on the implementation data.

## Results

From eight original KTAs, one declined to participate citing other work took priority then two left mid-project for other employment. Another KTA was employed and joined the co-operative inquiry at the beginning of Phase 2 making eight (or six at any one time) KTAs in total who participated in the co-operative inquiry. The KTAs agreed the replacement could join the group, and he did not upset the dynamics and contributed appropriately. Between three and six KTAs attended eleven KTA group meetings in total with HW. 20/24 interviews with CLs (7), ALs (7) and Ms (6) were carried out. Not all were available for interview because of maternity leave or relocation.

**Figure 1:**
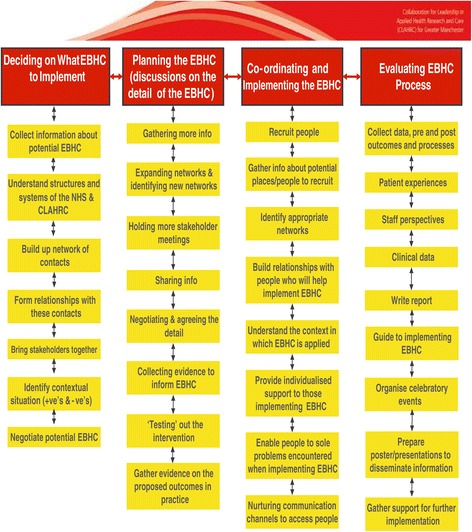
**Activities of the KTAs in the process of facilitation.**

Figure 1 depicts a conceptual model that emerged from the data analysis to answer the research question (the facilitation of the implementation of EBHC to improve patient care). Altogether, the KTAs facilitated their projects across numerous organisations (>20) working with people involved with commissioning and delivering healthcare. It was therefore facilitation on a large scale. The following section describes the KTAs experiences of facilitation (Figure 1) and shows the many complexities and difficulties that they encountered. In brief, the process began with the KTAs assisting with the decision on what EBHC to implement (for example, they identified appropriate individuals and groups and organised meetings so that they could be brought together to discuss the potential topic and actual evidence to be implemented). Then they passed through a planning phase where the exact detail of what was to be implemented was determined in negotiation with key stakeholders; this involved much to-ing and fro-ing between various people and groups. To the actual implementation of the EBHC which needed the KTAs to actively recruit people to be involved in implementation and, then, to coordinate and drive forward the implementation with those recruited. Finally, to evaluate the EBHC which required the KTAs to gather and analyse data. The different phases of facilitation are presented as such to show how the work of the KTAs gradually changed over time as the projects progressed. However, while this is presented linearly, it does not mean that the phases were passed through in orderly fashion. Movement between phases was iterative and quite often activities that began in the earlier phases continued all the way through, for example, the maintenance of relationships helped to make later activities run more smoothly

.

### Assisting with the decision on what EBHC to implement

Even though, at the beginning, the broad area for the implementation of EBHC was known for each project, there was a range of starting points for the KTAs. For example, in one project the focus was yet to be negotiated with key groups: commissioners, clinicians, patients, and representatives of other organisations in primary, secondary and community care. In contrast, in another project, the focus was already identified and agreed. This meant the KTAs spent different amounts of time assisting in the decision on what EBHC to implement. However, there was still a need in this phase for all KTAs to collect different types of information which would help confirm the decision for implementation including: epidemiological information on the local population, data on quality performance measures of primary care and community based health care organisations, patient perspectives, insider knowledge of how the organisation worked, processes of care and costs of the proposed EBHC. In situations where KTAs could not find this information out for themselves, they learnt to develop a network of contacts to liaise with for the necessary information.

The KTAs highlighted the importance of forming relationships with contacts to access the information they needed. Only one of the KTAs had prior primary care experience, which helped her on occasions to know where and who to access. However, for all the KTAs, at times, the lack of uniformity in the structure and operation of the NHS in general, the health care commissioning agencies, community based organisations, general practices and hospitals, made finding the correct people difficult.

Sometimes they resolved these difficulties by referring back to the CLs as they had pre-existing relationships with the people the KTA needed to access. For example, formal clinical networks enabled KTAs to gain access to suitable contacts and information. At other times, CLs used their position to gain access to executives at health care commissioning agencies and senior medical colleagues to find out their views and get sign off for projects.

An additional layer of complexity was added by GM CLAHRC itself, as it was not perceived to be an established part of the NHS. This resulted in NHS staff tending to be suspicious of requests for information and data, and who then delayed or refrained from providing requested information. This was despite the GM CLAHRC being a formal collaboration between a university and the NHS, with KTAs employed by a host NHS organisation. One KTA explains the process and frustrations in trying to get information:

… I think that because we are external to an extent even though we work for the NHS … so you have to then find the person to speak to, get them buying in and then ask them for information and then they request it from somebody else and then it is sent to them and back to you again, and it’s just so inefficient and it just makes a lot more sense if they just if they just give you access. [KTA 1]

The apparent inefficiency was said to slow down initial progression.

During this time, KTAs increased their knowledge of the evidence base about the disease condition and existing relevant guidelines on the topic of proposed projects. However, sometimes ideas for projects would change and KTAs would need to begin again collecting new evidence. There were several reasons for changes in the proposed topics, for example, if it were not a priority for influential clinicians and health care organisations.

At this time, different types of meetings were held with key groups. The purpose of these meetings were to: discuss the need for EBHC and potential implementation areas, share the evidence collected to date, suggest potential EBHC opportunities, garner support, further information, and identify drawbacks:

… so we had these meetings and asked people, we didn’t have any patients there but we had people involved in [disease area] from primary and secondary care and we asked them for their opinions on what kind of work we should do. So from that we had just like the first draft of ideas of where we should go with our project… [KTA 4]

Generally, the KTAs led these meetings alone then fed back to the team. In effect, the KTAs function was to pull disparate strands of information and people together to assist the CL and other stakeholders to make or confirm decisions about the focus of the project.

### Planning the EBHC

When the topic for implementation was agreed, the KTAs then gathered further information on the specifics of the proposed implementation. Information built on that collected in the previous phase and included finding out: (a) who and what competencies are needed to deliver the EBHC, (b) Which settings to select, (c) The resource implications, (d) Whether the project might fall under the auspices of clinical audit, (e) How they might determine the likely viability of the proposed EBHC in actual practice, (f) What support and formal education is required to deliver the EBHC, (g) What other agencies need to be engaged, (h) The degree of variation permissible in the application of the EBHC and (i) How the projects should be evaluated. The KTAs found that their contacts did not have the information that was now required so they had to extend their networks. In this phase, there was a great deal of ‘to-ing and fro-ing’ as knowledge and ideas were exchanged and decisions about the detail were made iteratively between KTAs, CLs, ALs and Ms and in partnership with stakeholders. They found this was time-consuming and painstaking.

During this time, the KTAs gathered a range of different types of evidence a) Research based articles, b) Reports from other implementation studies, c) Government policy or guidance documents, d) Interview/questionnaire data and e) Health databases. The KTAs and CLs were responsible for considering how these different types of evidence may be used to improve patient care. Apart from the instrumental use of evidence, reports, for example, were employed to inform conceptual ideas for the justification or necessity of projects [25]:

They (KTAs) went to other projects, they dug out you know already evidenced material if you like, they looked at policy documents but they looked at clinical documentation so they didn’t just read the evidence base that was offered to them if you like from the first PCTs [health commissioning agencies] setting up, they looked at other versions of this and what that meant was that when they went out to talk to the remaining, … so when they went out to the other three PCTs it meant that they didn’t only have to talk about the [name of PCT] experience which some people would have just rejected, they were able to go with a wider evidence base to talk about what other people had done, to talk about what kind of evidence there was, what kind of evaluation that was. They built their own credibility in part by being able to talk more broadly about that… [AL2]

In practice therefore there was a broad definition of what was counted as ‘evidence’ and how it was employed.

Sometimes, the evidence was lacking, especially with regards to the process of care, for example, one of the teams was developing a tool kit for stroke specialists and needed to gather evidence on what should go in it, who should deliver it, and where. This was because national policy documents had stipulated a clinical review was needed but had not said of what this should consist. The KTA carried out some interviews to overcome this lack of evidence. In another example, a KTA collected data to show a need for the EBHC:

Yes, so for example they [KTAs] did an audit of fifty something case notes and they could see going through them that there was actually no discharge plans at all, whereas when they actually spoke to the specialist nurses in the hospital they say “oh yes that gets done”, and it hadn’t happened with any of the ones they were looking at. [AL2]

This meant that the KTAs were not only facilitating the utilization of evidence but were generators of evidence too. In some projects, the KTAs tested whether the proposed EBHC was feasible and acceptable to general practices then made revisions to it.

### Coordinating and/or implementing the EBHC

To actually implement the EBHC across hospitals and numerous primary care and community based organisations, more information was sought by the KTAs. This time, it was to identify the right contacts who could give names of clinicians, general practices and services where implementation could take place. Even though they had previously developed supportive contacts and obtained ‘buy-in’ from senior staff, the relationships with consultants, general practitioners (GPs), specialist nurses, district nurses, receptionists and practice managers were mostly new.

The approach to recruitment of general practices was systematic to get a representative sample of the local population. In one project, recruitment was assisted by a local opinion leader who sent a letter to all potential participants. This opinion leader’s views were valued by his peers. In another case, an opinion leader actually recruited on behalf of a KTA. Word of mouth also helped in the recruitment of general practices.

Also significant in recruiting people, the KTAs negotiation and communication skills improved throughout the project:

I have improved my skills of negotiating with people, because like again in an informal way we have had, it has kind of trying to get people on board, so maybe communication skills in that respect have improved like dealing with people and working out what motivates them, what you need to say to them to try and get them to do the work for you and things like that. [KTA 10]

The data shows that KTAs acquired four key areas of knowledge that helped them to present reasoned arguments to people in an effort to recruit them. For example, they often referred to key stakeholder support to gain interest (Table 3). Building on the knowledge they had gained and on their past experiences, the KTAs would also adapt a variety of motivational techniques to influence people to participate (Table 4). This adaptability was an important skill as individuals had differing concerns about the proposed EBHC. This meant that KTAs were referring to perceived important social, clinical and local norms in order to recruit people [25].

**Table 3:** **Four areas of knowledge that helped KTAs recruit people for the implementation of EBHC**

**Four areas of knowledge that helped KTAs recruit people for the implementation of EBHC**	
1.	Knowledge that key stakeholders are supportive including commissioners
2.	Knowledge of the evidence, policy and/or guidelines that support the clinical aspects of the EBHC
3.	Knowledge of the performance of general practices in relation to the condition
4.	Knowledge of the condition including its diagnosis and treatment

**Table 4:** **Strategies employed in recruitment of practitioners and managers by KTAs**

**Strategies employed in recruitment of practitioners and managers by KTAs**	
1.	To find out how the evidence is interpreted and what kind of evidence, if at all would be influential
2.	To discuss potential cost-effectiveness of the EBHC project
3.	To indicate the project was part of a large scale National Institute of Health Research funded project
4.	To explain how the individual will gain personally
5.	To show how the project will fulfil a need for the practice/unit
6.	To demonstrate how it will benefit patients
7.	To indicate how it will bring income to the practice

All the tasks were project managed by the KTAs, CLs, ALs and Ms. However, each of the projects had different approaches to how they implemented EBHC in the clinical areas. The variation occurred in the formality of the approach, the level of prescription of the EBHC, what needed to change to achieve implementation and the amount of time KTAs spent in each setting where the EBHC was implemented. For example, in one project, an invitation about a planned audit was sent to general practices. Following the audit, written and oral guidance on how clinicians could implement EBHC was offered. This approach was more ‘hands off’ compared to another project in which a link person or team from each practice was identified through which the EBHC was implemented. The involved KTA describes the processes:

…and then once we had got practices interested, going and meeting with them and making sure that they were happy to be part of it, getting them to sign up and then it kicked off the real work of running workshops, going into practices and discussing the …work with them, collecting data, analysing data. Various things we did along the way with practices like assessing their progress against various different frameworks and scorecards and using that to help us think what we can do with them to help them improve more. [KTA 10]

In this approach, the KTAs spent a varied amount of time with each practice depending on how much support was required to implement the EBHC.

Some participants were able to implement the EBHC without much assistance but others found it difficult to move forward. These situations were a challenge for the KTAs. They, in consultation with CLs, ALs and Ms, took the line that they were there to help staff to implement the EBHC for which they had ‘signed up’. They were not in the position to tell them what to do or deliver tasks for them, but they tried to provide as much support as possible. The KTAs also found that honesty, directness and focussing on what needed to be done were effective in helping staff to persist. The KTAs tried to work in partnership with staff, maintaining good relationships with them, to secure commitment and loyalty to the project.

Contextual problems encountered by the KTAs included: (a) Poor internal communication channels, (b) Poor leadership, (c) Rejection of the value of using evidence to improve practice, and (d) Poor administrative/management systems. The KTAs, in discussion with the project team, took different approaches to the analysis and actions to overcome these difficulties. Some identified and acted on these issues informally, for example, by noting and addressing them where possible at subsequent meetings. In contrast, others took a formal approach, for example, through supporting staff to use specific tools that enabled them to review and change their situation to enable the implementation of EBHC [26]. Altogether, this shows that an ability to mediate contextual issues with participants was an important part of facilitation for the KTAs.

### Evaluating EBHC

While the evaluation of EBHC projects did not unfold sequentially after the implementing and co-ordinating phase, it was the concluding work of facilitation for all the KTAs before the projects coalesced and/or transformed into other projects. The KTAs often set up new systems to collect data because the information already gathered by general practices was not always appropriate for the purposes of evaluating the project. One KTA reports on the difficulties they have had getting suitable data:

We have had huge amounts of problems trying to get accurate data, even on some of them. There are some things that the practices measure for QOF [quality and outcomes framework] which is how they get paid, so they have got all their measures accurate for that but there are quite a few things which are measures we just said we would like to look at, and some of them we are still not getting accurate. We have been working on it for eight months and we still don’t get accurate data on it… and the computer systems don’t work and they won’t allow you. [KTA 5]

The KTAs had to enhance their understanding of databases to obtain this type of information. All the KTAs had access to academic advice about the evaluation of EBHC and drew upon research skills, although, they were not doing research, during this time.

KTAs analysed patient outcomes, and evaluated patient and staff experiences by individual or focus group interviews. In all but one project, with the benefit of hindsight, more and different types of data, for example, clinical data, could have been collected. The KTAs also collected data and information to develop general guides to support the spread of the project to new sites.

## Discussion

Our research draws on the experiences of 8 KTAs who were responsible for the facilitation of the implementation of EBHC across six projects in the GM area over one year. It is of sufficient scale to provide indicative findings of the process, methods and styles of facilitation that were employed. Figure 1 provides detailed information of the activities of the KTAs during each phase. Even though we did not set out to employ Ward et al’s framework for knowledge brokering, there are similarities in the work of their knowledge brokers and the KTAs [27]. They argue that knowledge brokering is neither a linear or cyclical process but one in which the various components and their corresponding activities interact over time [28]. Similarities can also be observed between the activities of the KTAs and another taxonomy of facilitation strategies that was developed from a systematic review of the process of facilitation in research utilisation in nursing [8]. There were also resemblances to strategies for the implementation of clinical guidelines [29,30]. Further research is required, therefore, to elucidate whether the activities of facilitation in practice is actually the same regardless of whoever implements EBHC and the underpinning theoretical perspective. This indicates the importance of clearly defining the role and activities for facilitation in research for the purposes of comparability.

Although, the activities of facilitators appear similar, the findings show that in practice variations existed between projects in the length of time of each phase, who was involved, level of prescription of the method for the management of change and fidelity to the interventions i.e. how much adaption occurred in each context. The reasons for this depended on the EBHC to be implemented, for example, whether it was a technology or administrative process, available resources, personal preferences of the project team and participants and the context in which it was to be implemented. This means that only a skeletal process framework for facilitation of the implementation of EBHC can be offered, that is, ‘no one size fits all’.

The findings also support the conceptualisation of the purpose of facilitation as a continuum ranging from a task oriented approach where the focus is on achievement of a goal to a holistic approach where the emphasis is on helping and supporting individuals and/or teams to improve their way of working [6]. These differing foci were in use simultaneously during our project. For example, project management which aimed to achieve specific implementation goals and team-building exercises to promote effective working in GPs ran alongside one another.

Underlying assumptions about the process of implementation tend to be portrayed from either a rational-technical or interactive social perspective [27]. However, presenting the process from one or the other perspective may obscure what happens in practice [31]. The data presented here indicate that neither a purely rational-linear or interactive perspective underpinned the implementation of EBHC. An interactive approach was demonstrated by KTAs, for example, when they not only appealed to the logic of the research but referred to the values and beliefs of the audience and used their own credibility to gain agreement for EBHC among commissioners [32]. This approach draws on constructionist epistemology that states that knowledge is co-created between people, is value laden and is contested [31]. The interactive model also features to some extent in the work of major producers of clinical guidelines including the Guidelines International Network and National Institute for Health and Care Excellence, UK [33,34].

The interactive approach was also apparent when practitioners did not just implement EBHC because of the strength of the research argument, but needed to situate it in their own context to suit their and their patients’ needs [35]. Thus, research was not always ‘ready to use’, did not take into account all the variables with which people need to be aware and had to be shaped into something practically useful by the KTAs [35]. The process of implementation was therefore, dynamic, proactive, and contextual which is reflective of an interactive model [31].

Our projects also had elements that map onto a rational-linear approach to EBHC. Rational-linear proponents tend to assume there is a unidirectional process from research to implementation without local adaptation. Within this approach, there is a tendency to assume passive dissemination will lead directly to implementation [31]. A rational-linear approach was found in our projects where part of the implementation strategies required fidelity to the EBHC and where dissemination strategies were web-based. Altogether, this reveals that in our project taking either a rational linear or interactive approach to the implementation of EBHC was insufficient and a framework which is inclusive of both approaches was helpful in explaining the implementation of EBHC [31].

Figure 1 provides empirical evidence for the Knowledge-to-Action cycle as a way to operationalise implementation [30]. Knowledge-to-action concepts including identifying the knowledge to action gaps, adapting knowledge to local context, selecting, tailoring and implementing the intervention, monitoring knowledge use and evaluating outcomes are recognisable in the experiences of implementation of the KTAs [30]. This framework is particularly useful as it recognises that facilitators can be generators of data, for example, through carrying out audits to clarify the problem locally as well as assisting in the implementation of externally produced evidence. The evidence also reveals the iterative nature of the process in which there was an ‘eye’ to the next step where imagination or testing of what was to come in the next stage was included in the decisions.

In our study, the KTAs lacked contextual knowledge of the NHS which was not helped by the lack of uniformity between NHS Trusts and general practices in terms of how they were structured, managed and the services they provided. They had to discover this to understand what was likely to be feasible and how to gain agreement about the EBHC. The acquirement of contextual knowledge through networking and gaining information was a significant and explicit part of defining and planning for a realistic and successful implementation of EBHC. In local, insider implementation studies, the need for networking and understanding organisational processes and culture is still important but may be more likely to be known to those facilitating the project and, therefore, be on a lesser scale and less likely to be reported.

As our study was large-scale, and centrally driven by the KTAs, CLs, ALs, and Ms [36], one activity of the KTAs was to recruit individuals or groups of people to participate in implementing EBHC. While some of these people may have been involved in the early negotiations over what to implement, the majority were not. In this instance, defining and agreeing on the problem occurred at two time points at the beginning and during the implementation phase. This additional activity is often not represented in frameworks of EBHC because they are targeted at the level of the practitioner as it is anticipated that the people who define the problem are the ones who will implement it [37]. This also meant that opinion leaders [38,39] were recruited at two different time points and were not always the same person: first, to help with the negotiation and communication of the EBHC to be implemented and, second, to support the recruitment of general practices and practitioners.

Another recruitment strategy of some KTAs was to employ personal or financial incentives. This strategy of recruitment echoes Roger’s notion of relative advantage where potential adoptees of an innovation assess whether to uptake an innovation according to whether it is perceived as bringing better rewards economically or in terms of social prestige than the current arrangement [35]. Incentives for promoting the spread of EBHC require further research.

A clear understanding of the process and possessing the right skills are requisites for successful facilitation [6,40]. In our case, the KTAs had a range of prior facilitation experiences: some had carried out facilitation in commercial organisations, another in the NHS as a senior community nurse whereas others had little experience. However, after two years, the KTA’s had acquired a better understanding of their role and the process, and become largely independent. They had refined many skills including taking an honest and direct approach which focussed on the task in hand. These findings build on previous work illustrating how facilitators develop their skills and confidence over time [41]. They benefited from education and mentoring, both prior and alongside the project in: (a) Change management, (b) Project management, (c) Interpersonal communication skills, (d) The care and organisation of patients with the disease being targeted for improvement and (e) Research skills especially in methods of evaluation. Given the scope and range of skills required, it appears that when implementing EBHC at commissioning and provider levels, facilitation should be perceived as a full-time role in itself. More work is required to understand how facilitators can be more formally supported and educated, thus increasing the numbers of people capable of operating at this level and further enabling the implementation of EBHC. Other inquiries are needed to explore how more experienced teams of facilitators consolidate and enhance their practice within a changing context of commissioning in primary and community care [42].

The KTAs were perceived as coming from a ‘new and strange organisation’ to the NHS participants with whom they had to work and all except one began with experience external to the clinical area. Latterly, practitioners from primary or acute sector were seconded to either be KTAs for specific projects or to provide specialist clinical knowledge. The gain is thought to be in three ways: i) as they will develop their skills and knowledge base in facilitating EBHC, ii) the GM CLAHRC will benefit from their additional clinical skills and knowledge and understanding of local context and iii) it serves to raise awareness of GM CLAHRC and how it works with the NHS. Their involvement will also increase capability in the local workforce regarding the implementation of EBHC.

The co-operative inquiry provided a forum in which the KTAs could discuss openly their experiences and reflect critically upon the process of facilitation. This provided a learning environment that expanded their experience and deepened their understanding about facilitation, which they were able to articulate clearly to others. This was in addition to the formal education they received. The co-operative inquiry itself ran relatively smoothly; this was because the KTAs negotiated time away from daily activities to attend. In comparison, the actual process of implementation, as shown, was messy, complicated and difficult to plan far ahead.

### Limitations

Direct observations of the KTAs in their daily work would have provided a more detailed picture of how they facilitated the implementation of EBHC. However, in doing the co-operative inquiry, they ‘observed’ themselves and while they could be accused of bias, we deliberately took a critical position with individual accounts during the group meetings. A strength of co-operative inquiry is the personal development which occurs from participants’ direct involvement in the research but at the same time this feature may be construed as a disadvantage as biases may go unnoticed even though the process is critical. In our study, to counter this effect, preliminary data analysis from the interviews with the CLs, ALs and Ms were fed back to the KTAs and then in turn the results of the overarching analysis were fed back to CLs, ALs and Ms during the second round of interviews and some of them were involved in the writing of the paper (RB and GH) with the KTAs.

Two KTAs left their employment, which could have disrupted the research and affected the quality of data collected. However, the disruption was minimal because the new KTA took over early in the process during phase 2. The allocation of KTA work also meant that they overlapped one another having a primary and secondary role in at least two of the projects so that no project was left bereft of a KTA even when KTAs left.

The study was not designed to demonstrate whether facilitators of the implementation of EBHC contribute to improved patient or health outcomes. Further research is required to investigate the link between the two and indeed if different facilitation approaches have different impacts on outcomes, within the knowledge that context is a key influence on the facilitation process itself.

## Conclusion

Our research is important because it articulates insider accounts of the actual process of facilitation and the activities involved in the implementation of EBHC at both commissioning and provider levels. The KTAs who were external to the institution in which EBHC was implemented, were embedded in an organisation (GM CLAHRC) and supported by a team of people who had different amounts of input and expertise. A diverse and flexible approach to the implementation of EBHC is required depending on the context where it is to take place. There was considerable lack of uniformity in the organisation of the health care systems, which meant, for example, that one method of communicating with staff in one setting might not be appropriate in another. The iterative processes in the Results also provide evidence that contextual analyses are required to work successfully with stakeholders. Insider knowledge of structures, interactions between stakeholders and patient services made facilitation easier. If the KTA did not already possess this knowledge then time was spent acquiring it. Altogether this research demonstrates in some detail how no single method of facilitation will fit all circumstances. Further investigation is required of how personal and financial incentives may play a part in gaining ‘buy-in’ of participants for the implementation of EBHC. The focus of EBHC is about the implementation of research, which is usually externally generated, but in this study the KTAs were also responsible for generating evidence to show there was an issue or to solve a problem. The findings are relevant to policy makers as they suggest that facilitation in this capacity is a dedicated role especially since the scope and skills required are extensive. Formal education and support systems are required to prepare a cadre of facilitators capable of operating at this level. Further prospective research is required to demonstrate how facilitation of the implementation of EBHC affects patient health outcomes.

## Abbreviations

ALs: Academic leads
CLs: Clinical leads
Ms: Managers
EBHC: Evidence based health care
GM CLAHRC: Greater Manchester collaboration for leadership in applied health research and care
KTA: Knowledge transfer associate
NHS: National Health Service
UK: United Kingdom
